# Penile Tourniquet Injury in a Patient With Schizoaffective Disorder Presenting With Altered Mental Status

**DOI:** 10.7759/cureus.106636

**Published:** 2026-04-08

**Authors:** Marino A Kokolis-Lattanzio, Justin Salvesen

**Affiliations:** 1 Urology, Touro College of Osteopathic Medicine, Middletown, USA; 2 Psychiatry, Garnet Health Medical Center, Middletown, USA

**Keywords:** command auditory hallucinations, genitourinary trauma, penile tourniquet, rare cause of altered mental status, schizoaffective disorder, self-inflicted injury, stroke mimic, urologic emergency

## Abstract

Penile tourniquet syndrome is a rare urologic emergency that can lead to ischemia, necrosis, and long-term functional impairment if not promptly recognized and treated. We present the case of a 52-year-old incarcerated male with a history of schizoaffective disorder who presented with altered mental status initially concerning for an acute cerebrovascular event. Further evaluation revealed a self-inflicted penile tourniquet using a sock in the setting of command auditory hallucinations. The patient developed significant penile edema, ischemic changes, and urinary retention requiring catheterization. Despite initial improvement with conservative management, follow-up demonstrated tissue sloughing of the glans with concern for long-term reconstructive needs. This case highlights the importance of thorough physical examination in patients with altered mental status, the role of psychiatric illness in self-inflicted injury, and the potential for delayed complications even with early intervention.

## Introduction

Penile tourniquet syndrome is an uncommon but potentially devastating condition caused by circumferential constriction of the penis, leading to impaired venous and lymphatic drainage, followed by arterial compromise and tissue ischemia. If not promptly addressed, it may progress to necrosis, urethral injury, or even amputation. Etiologies include accidental hair tourniquets, constrictive devices used for sexual purposes, and, less commonly, self-inflicted injury in patients with psychiatric disorders.

Patients with severe psychiatric illness may engage in self-harm behaviors under the influence of delusions or command hallucinations, posing unique diagnostic and management challenges. Additionally, altered mental status in such patients often prompts evaluation for neurologic emergencies, which may delay recognition of concurrent traumatic or ischemic processes.

We present a case of penile tourniquet injury in a patient with schizoaffective disorder presenting primarily with altered mental status, highlighting diagnostic complexity, multidisciplinary management, and delayed tissue complications despite initial conservative treatment.

## Case presentation

A 52-year-old incarcerated male with a past medical history of hypertension, coronary artery disease, schizoaffective disorder with bipolar features, seizures, prior subdural hematoma, and endocarditis was brought to the emergency department for evaluation of altered mental status. The patient was last known to be well earlier in the day and was subsequently found minimally responsive with garbled speech and a history of multiple recent falls. He was a poor historian upon presentation.

On arrival, the patient was afebrile with a blood pressure of 164/90 mmHg, a respiratory rate of 21 breaths per minute, and an oxygen saturation of 98% on room air. He appeared lethargic but was arousable to verbal stimuli. Neurologic examination revealed diffuse weakness without focal deficits, and baseline anisocoria was noted.

Laboratory evaluation demonstrated acute kidney injury (creatinine 2.03 mg/dL; baseline approximately 1.0 mg/dL), elevated troponin (42 ng/L), and a supratherapeutic valproic acid level of 114 µg/mL. Creatine kinase was 283 U/L, and lactate was 1.9 mmol/L (Table 1). The patient’s home medications included valproic acid, quetiapine, and other sedating and anticholinergic agents, which were suspected to contribute to his toxic-metabolic encephalopathy. A stroke alert was activated, and computed tomography of the head and computed tomography angiography of the head and neck were negative for acute intracranial pathology or large vessel occlusion.

**Table 1:** Laboratory findings on admission Summary of laboratory values obtained on presentation, highlighting acute kidney injury and supratherapeutic valproic acid levels contributing to toxic-metabolic encephalopathy.

Test	Result	Reference range	Interpretation
Creatinine	2.03 mg/dL	~0.6-1.3	Acute kidney injury
Troponin	42 ng/L	<14	Demand ischemia vs. injury
Creatine kinase	283 U/L	20-200	Mild elevation
Valproic acid	114 µg/mL	50-100	Supratherapeutic
Lactate	1.9 mmol/L	<2	Normal

Neurology evaluation determined that the patient’s presentation was most consistent with toxic-metabolic encephalopathy in the setting of supratherapeutic valproic acid levels and polypharmacy, rather than a primary neurologic or psychiatric cause. Valproic acid and other contributing medications were held, resulting in gradual improvement in mental status.

During a comprehensive physical examination, a tube sock was discovered tightly constricting the mid-shaft of the penis, functioning as a tourniquet. The constricting material was removed at the bedside. Examination revealed significant distal penile edema, erythema, tenderness, blistering, and areas of skin sloughing, concerning for evolving ischemic injury. Upon further evaluation after improvement in mental status, the patient reported that the behavior was driven by command auditory hallucinations.

Urology was consulted. Bladder scan demonstrated urinary retention exceeding 1100 mL, and a Foley catheter was successfully placed with return of approximately 1400 mL of urine. The patient was subsequently admitted for management of acute encephalopathy and penile trauma. A summary of the patient’s hospital course and key clinical events is outlined in Table 2.

**Table 2:** Timeline of clinical course Chronological summary of key clinical events from initial presentation through hospitalization and follow-up, illustrating diagnostic evaluation, therapeutic interventions, and progression of penile injury. AMS: alterted mental status

Hospital day	Event
Day 0	Presentation with AMS, stroke workup negative
Day 1	Penile tourniquet identified and removed
Day 1-2	Foley placement, urinary decompression
Day 2-5	Mental status improves after medication adjustment
Discharge	Stable with Foley, outpatient urology follow-up
Follow-up	Tissue demarcation and glans sloughing noted

Psychiatry was consulted during hospitalization. The patient’s medication regimen was reviewed in the context of supratherapeutic valproic acid levels and polypharmacy. Adjustments were made to reduce sedating and anticholinergic burden while addressing underlying psychotic symptoms. The patient was monitored for ongoing hallucinations and safety risk. Prior to discharge, safety planning was completed, and arrangements were made for continued psychiatric care within the correctional healthcare system.

Over the course of hospitalization, the patient’s mental status improved significantly, and he became alert and interactive. Urologic and wound care teams closely monitored the penile injury. Initial concern for ischemia was noted due to marked edema, bullous changes, and superficial tissue erosion.

Following improvement in his encephalopathy, the patient became more alert and was able to participate in further evaluation. He reported that his behavior was driven by command auditory hallucinations instructing self-harm. While a comprehensive formal mental status examination was not fully documented in the available records, the patient demonstrated impaired insight and judgment in the context of ongoing psychotic symptoms. His thought process appeared goal-directed, though influenced by these hallucinations. No active suicidal or homicidal ideation was reported at the time of reassessment.

With conservative management including local wound care and urinary decompression, penile swelling decreased, and tissue changes began to demarcate without early evidence of full-thickness necrosis. The patient was discharged in stable condition with a Foley catheter in place and plans for close outpatient urologic follow-up.

At follow-up evaluation, progression of injury was noted with sloughing of distal glans tissue and continued demarcation. Although surgical intervention was not immediately required, there was concern for long-term tissue viability and potential need for reconstructive management at a tertiary care center.

## Discussion

Penile tourniquet syndrome results from circumferential constriction that impairs venous and lymphatic outflow, leading to progressive distal edema and eventual arterial compromise. This may result in ischemia, necrosis, urethral injury, and, in severe cases, penile amputation [1-4].

This condition is most commonly described in pediatric populations, particularly in association with hair tourniquets [1]. In adults, reported etiologies are less frequent and typically involve constrictive devices used for sexual purposes or, less commonly, self-inflicted injury [2-4]. Cases arising in the context of psychiatric illness are rare, and those specifically associated with command auditory hallucinations are exceedingly uncommon, highlighting the unique nature of this presentation [5,6].

This case is notable for several reasons, including its atypical presentation and underlying psychiatric etiology. First, the patient presented with altered mental status primarily attributed to toxic-metabolic encephalopathy in the setting of supratherapeutic valproic acid levels and polypharmacy, prompting activation of a stroke alert, illustrating how penile tourniquet injury may be easily overlooked when initial evaluation is focused on neurologic emergencies. These findings emphasize the importance of considering medication-related causes in patients with psychiatric comorbidities [5].

Second, this case highlights the role of psychiatric illness in self-inflicted genitourinary trauma. Command auditory hallucinations are a well-recognized feature of psychotic disorders and are associated with an increased risk of self-injurious behavior, particularly in patients with impaired insight [5,6]. This underscores the importance of maintaining a high index of suspicion for occult injury in patients with severe psychiatric disease presenting with altered mental status.

Early identification and removal of the constricting device remain the cornerstone of management and are critical in preventing progression to irreversible ischemia [2-4]. In this case, prompt bedside removal and conservative management, including urinary decompression and local wound care, initially resulted in clinical improvement with reduction in edema and stabilization of tissue viability.

However, this case also demonstrates that delayed complications may occur despite early intervention. The subsequent development of tissue sloughing and progressive demarcation of the glans suggests ongoing ischemic injury, likely related to the duration and severity of initial constriction [1,4]. This highlights the importance of close outpatient follow-up, as initial improvement does not preclude later deterioration or the need for reconstructive intervention.

Finally, this case emphasizes the importance of thorough physical examination in patients presenting with altered mental status. While neurologic etiologies must be rapidly assessed and excluded, failure to identify concurrent traumatic or ischemic processes may delay critical interventions and worsen outcomes. A multidisciplinary approach involving emergency medicine, neurology, psychiatry, urology, and wound care is essential in optimizing both immediate and long-term patient outcomes [7].

## Conclusions

Penile tourniquet injury is a rare but potentially severe condition that may be associated with psychiatric illness and self-harm behaviors. This case underscores the importance of thorough physical examination in patients presenting with altered mental status, particularly in high-risk populations. While early intervention may mitigate severe complications, delayed tissue injury can still occur, necessitating close follow-up and potential reconstructive management. Increased awareness of this condition and its evolving clinical course is essential to optimize timely diagnosis and patient outcomes.
